# The Importance of Imprinting in the Human Placenta

**DOI:** 10.1371/journal.pgen.1001015

**Published:** 2010-07-01

**Authors:** Jennifer M. Frost, Gudrun E. Moore

**Affiliations:** Clinical and Molecular Genetics Unit, Institute of Child Health, University College London, London, United Kingdom; University of Cambridge, United Kingdom

## Abstract

As a field of study, genomic imprinting has grown rapidly in the last 20 years, with a growing figure of around 100 imprinted genes known in the mouse and approximately 50 in the human. The imprinted expression of genes may be transient and highly tissue-specific, and there are potentially hundreds of other, as yet undiscovered, imprinted transcripts. The placenta is notable amongst mammalian organs for its high and prolific expression of imprinted genes. This review discusses the development of the human placenta and focuses on the function of imprinting in this organ. Imprinting is potentially a mechanism to balance parental resource allocation and it plays an important role in growth. The placenta, as the interface between mother and fetus, is central to prenatal growth control. The expression of genes subject to parental allelic expression bias has, over the years, been shown to be essential for the normal development and physiology of the placenta. In this review we also discuss the significance of genes that lack conservation of imprinting between mice and humans, genes whose imprinted expression is often placental-specific. Finally, we illustrate the importance of imprinting in the postnatal human in terms of several human imprinting disorders, with consideration of the brain as a key organ for imprinted gene expression after birth.

## Introduction

Pronuclear transfer experiments in mice in the early 1980s showed that maternal and paternal genetic contributions were non-equivalent and that both were indispensable for normal development [1], [2]. The introduction of reciprocal translocations into mice, creating regions of uniparental disomy, showed that discrete areas of the mouse genome were subject to differential parental regulation [3]. In parallel with this fascinating mouse work, it was observed that several non-Mendelian human syndromes showed similar inheritance to phenotypes seen in the disomic mice [4]. The mapping of deletions causative in Prader Willi (PWS) and Angelman (AS) syndromes, for example, permitted localisation of parentally non-equivalent genomic regions in humans [4]. In 1991, the first endogenous imprinted genes were identified [5]–[7]. This parent-of-origin, monoallelic gene expression, with its associated differential DNA methylation (first shown in 1993, [8]) became defined as genomic imprinting.

Genomic imprinting, found predominantly in eutherian mammals, is an epigenetic phenomenon whose evolution may be linked to a dichotomy between paternal and maternal resource allocation. This is potentially powerful enough to promote evolution of unequal gene expression between selected parental alleles. Parental-specific monoallelic expression thus balances fetal growth to the equal benefit of both parental genomes, in spite of the resulting potentially damaging haploinsufficiency [9].

The canonical example of allelic expression of imprinted genes balancing growth is evident with the paternally expressed *Igf2* and maternally expressed *Igf2r* genes [5], [7], [10]. *Igf2* is a potent enhancer of fetal growth and inappropriate expression disturbs normal growth in mice [10]. A reduction in *Igf2* expression leads to growth restriction, whereas biallelic expression and the subsequent increase in the number of *Igf2* transcripts leads to overgrowth [11], [12]. Maternally expressed *Igf2r* has the opposite effect on growth, as the Igf2r protein acts as a negative regulator of Igf2 by binding to the Igf2 protein, reducing its bioavailability and targeting it for lysosomal degradation [13]–[16]. Monoallelic expression of imprinted genes is controlled by allelic DNA methylation, added differentially to the imprinting control regions (ICRs) of parental germlines [17], [18]. The paternal allelic expression of murine *Igf2* is also found in humans, and in both species monoallelic expression is mediated in cis by maternal DNA methylation at the H19 ICR, the differentially methylated domain (H19 DMD) [19]–[22].

*IGF2* is also an important growth enhancer in humans, and its expression and subsequent phenotypic effects may be similarly impacted by dysregulation of imprinting. A loss of methylation at the H19 DMD in humans is found in a subset of Silver Russell syndrome (SRS) cases [23]. The main phenotype of SRS is severe intrauterine growth restriction (IUGR) that could be caused by a reduction in *IGF2* transcription as a result of a loss of methylation at the H19 DMD [23]. Hypermethylation at the H19 DMD is found in 30% cases of Beckwith Wiedemann syndrome (BWS) [24], and the overgrowth macroglossia and organomegaly associated with this disorder may be caused by an increase in *IGF2* transcription as a result of its biallelic expression. *IGF2R* imprinting in the human, in contrast, is polymorphic, rare, and most likely restricted to the placenta [25]. Recent evidence of a potential human orthologue of the murine ncRNA *Air*, responsible in the mouse for paternal *Igf2r* silencing, indicates that some key features of reciprocal murine *Igf2/Igf2r* imprinting may be present in humans [26].

## Human Placental Development

**Figure 1:**
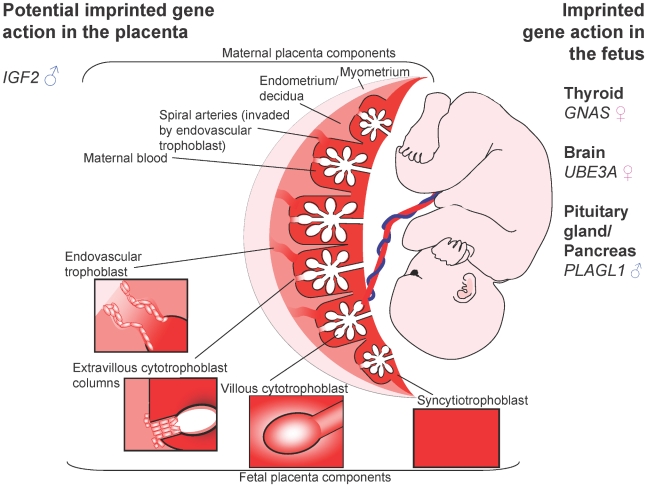
The human fetus and placenta. Villous trophoblasts of the human placenta grow as a branched structure, maximising exchange with maternal blood. Extravillous trophoblast invade into the maternal endometrium, and some cells colonise maternal spiral arteries, expanding them to maximise blood flow. ♂ = Paternally expressed; ♀ = maternally expressed. Imprinted genes are important during fetal growth. Some, such as *GNAS*, *UBE3A*, and *PLAGL*, have physiological impact on the fetus only. Other genes may influence growth in utero via the placenta, or the fetus and placenta. The IUGR seen in SRS, and overgrowth in BWS are suggestive of a role of *IGF2* in the human placenta.

The placenta, particularly the invasive trophoblast lineages, is an important focus for potential parental conflict. It is directly responsible for bringing maternal and fetal blood supplies into contact, facilitating nutrient exchange and determining resource allocation (Figure 1) [27].

Human embryos implant interstitially in a highly invasive manner. Leading edge trophoblast cells fuse to form a syncytium, resulting in a two layered structure of multinucleated syncytiotrophoblast and cellular cytotrophoblast. Protusions of syncytiotrophoblast interdigitate into the decidualised endometrium, forming contacts with the maternal blood supply (Figure 1). Extravillous cytotrophoblast, which may be analogous to the endoreduplicated murine giant cells, form columns from the tips of anchoring villae, attached to the basal plate, and extend through the syncytium. Invasive cells break away from these columns and migrate to colonise maternal spiral arteries. Interstitial trophoblast cells invade to expand the placenta from its edge outwards [28]. Invasion is partly controlled by the decidua, which expresses proteins, including a wide variety of IGF binding proteins, balancing invasion and fetal provision [29], [30]. Perturbation of this is evident in ectopic pregnancy, when invasion is far more extensive in the absence of the decidua [31].

## Genomic Imprinting in the Human Placenta

The physiological importance of genomic imprinting in humans can be demonstrated by the diseases resulting from mutations or epimutations in imprinted genes. Human imprinting disorders are somewhat rare but comprise a large group of diverse pathologies, primarily involving growth or neurological development. Consistent with the growth phenotypes observed, many of the imprinted genes known to-date are expressed in the human placenta (Table 1) [32], [33].

**Table 1:** Imprinted genes expressed in the human placenta, with phenotpyes associated with loss of gain of expression, where reported.

Locus	Gene	Active allele	Phenotype if biallelic or overexpressed	Phenotype of loss of expression/deletion/mutation
1p36	*TP73*		LOI in normal placenta	
6q24	*PLAGL1* *	P	TNDM (pUPD Hsa6q24, hypomethylation of ICR)	Reduced expression in IUGR
7p12	*GRB10* *	P (B) M (T)	SRS subset [mUPD7, linkage only]	Murine KO exhibit disproportionate fetal and placental (labyrinth) overgrowth
7q21	*TFP12*	M	None reported	
	*SGCE*	P		Myoclonus dystonia
	*PEG10* *	P	Hepatocellular carcinoma, linked to IUGR	Murine KO lacks spongiotrophoblast
	*PPP1R9A*	M		Murine KO abnormal dopaminergic signalling
7q32	*MEST Iso1* *	P		SRS subset (mUPD7, linkage only) No mutations found. Murine KO pre- and postnatal growth restriction
	*MEST Iso2* *	P		
	*MESTIT1* *	P		
	*KLF14*	M	None reported	
11p15	*H19* *	M	SRS	BWS
	*IGF2* *	P	BWS–Wilm's+other tumour development	SRS
	*IGF2AS*	P	None reported	
	*INS*	P		Permanent Neonatal Diabetes
	*KCNQ1* *	M		BWS+Long QT Syndrome 1
	*KCNQ1OT1* *	P	BWS	
	*CDKN1C* *	M		BWS – abdominal wall defects
	*SLC22A18* *	M		Unknown but within BWS region
	*SLC22A18AS* *			Unknown but within BWS region
	*PHLDA2* *	M	Decreased birth weight, possible IUGRMurine overexpression inhibits labyrinth and spongiotrophoblast growth	Increased birth weight+within BWS linked regionMurine KO show placental hyperplasia, specifically spongiotrophoblast
14q32	*DLK1* *	P	BWS subset. Murine transcgene overexpression of *Dlk1* results in high birth weight but a failure to thrive [102].	Murine KO show fetal growth restriction, postnatal catch-up growth and increased adiposity in adults on high fat diet [103]
15q11	*SNRPN*	P	None reported	
16p13	*ZNF597*	M	None reported	
19q13	*ZNF331* *	M		Reduced expression found in IUGR
	*PEG3*	P		Ovarian tumours and gliomaMurine KO adults display aberrant maternal care (females) and sexual behaviour (males)
	*ZIM2*	P	None reported	
20q13	*GNAS XL* *	P		Albright's hereditary osteodystrophy
	*GNAS Exon 1A* *	P		Murine KO suckle poorly, lean and growth restricted with increased insulin sensitivity
	*NESP*	M		Linked to pseudohypoparathyroidism Type 1bMurine KO react aberrantly to novelty

*Genes that when mutated/epimutated are associated with a human growth phenotype. Murine phenotypes are also shown where knock-out (KO) models have been created. UPD, uniparental disomy; TNDM, transient neonatal diabetes mellitus; SRS, Silver Russell Syndrome; BWS, Beckwith Wiedemann Syndrome; P, paternal; M, maternal; B, brain; T, trophoblast. http://igc.otago.ac.nz/home.html; [33].

Disease pathologies resulting from inappropriate imprinted gene expression may each be due in part, or completely, to an aberrantly functioning placenta. The placenta is fundamental to fetal growth, and Table 1 highlights the imprinted genes expressed in the placenta that have been implicated in fetal growth disorders. As previously stated, IUGR is a defining characteristic of the imprinting disorder SRS. Up to half of all SRS cases may be caused by a reduction in *IGF2* expression, as outlined above, but in the remainder the cause is unknown [23]. Whilst IUGR is often idiopathic, it is commonly accompanied by reduced blood flow through the placenta and limited invasion of the decidua and maternal blood vessels [34]. This phenotype is consistent with either the loss of expression of an imprinted gene involved in maximising recruitment of maternal resources (i.e., a paternally expressed gene), or an increase in expression of an imprinted gene acting to limit maternal input (i.e., a maternally expressed gene). A second disease associated with reduced placental perfusion is preeclampsia, whose matrilineal inheritance pattern has highlighted the possibility that imprinted genes might involved in its pathogenesis [35], [36]. In a recent study of 96 cases of BWS, seven resulted from maternally inherited *CDKN1C* mutations and of these, three pregnancies were complicated by preeclampsia, compared to three of the 89 BWS cases not related to *CDKN1C* mutations [37]. Interestingly, transgenic mice whose litters carry mutations of the maternal *Cdkn1c* copy display preeclampsia-like features, including hypertension, proteinuria, and abnormal trophoblast proliferation [38], [39]. These data suggest an important role for *CDKN1C* in a subset of preeclampsia cases, however, other imprinted susceptibility loci for this complication of pregnancy remain elusive [40].

The imprinted gene *PHLDA2* on human Chromosome 11 (Hsa11) is expressed predominately in the placenta, and its expression in the placenta at term correlates negatively with fetal birth weight [41]. Given that *PHLDA2* is maternally expressed, this trend is consistent with the parental resource conflict theory, that maternally expressed genes act to limit maternal resource provision. Further evidence that *PHLDA2* expression levels in the human placenta might be important in regulating fetal growth comes from two studies comparing placentas from normal and IUGR pregnancies. Both studies found higher levels of *PHLDA2* expression in placentas from IUGR pregnancies than placentas from non-IUGR pregnancies [33], [42].

Paternally expressed *MEST* is thought to play a role in angiogenesis in human trophoblast tissue and decidua, is highly expressed and robustly imprinted in the placenta [43]. *MEST* is located on Hsa7, and maternal uniparental disomy (mUPD) 7, is implicated in 7–10% of SRS cases. Additionally, one SRS case has been reported with a segmental mUPD for 7q31-qter, specifically implicating the *MEST* imprinting cluster in this instance, rather than any of the other imprinted genes on Hsa7 [44]. Currently, no direct evidence exists to link human *MEST* with disease, but mice deficient in *Mest* are pre- and postnatally growth restricted [45].

*GRB10* is a growth factor binding protein, maternally expressed specifically in cytotrophoblast and biallelic elsewhere, located on Hsa7 [46]. The mUPD7 implicated in 7–10% SRS cases would lead to biallelic expression of *GRB10* and may be linked to the growth restriction characteristic of SRS for this subset of patients. Currently, however, no evidence exists to directly link *GRB10* expression levels with growth in humans [47], [48]. In mouse embryos, *Grb10* is widely expressed from the maternally derived chromosome, and ablation of this copy causes embryonic overgrowth, such that neonates are 30% larger than wild-type littermates at birth [49]. This is accompanied by disproportionate overgrowth of the placental labyrinth [50].

## Imprinting in the Mouse Placenta

Further clues regarding a role for imprinted genes in the placenta have been derived from studies in transgenic and knockout mice. Ablating the expression of individual imprinted genes leads to a range of pathologies, depending on the gene. Murine paternally expressed *Igf2* has been shown to promote placental growth (see below), and loss of *Mest* or *Peg3* causes placental growth restriction. Conversely, deletion of maternally expressed *Igf2r*, *Cdkn1c*, or *Phlda2* results in placental hyperplasia [32].

The importance of genomic imprinting specifically in the murine placenta can be illustrated by the expression pattern of paternally expressed *Igf2*. Human and mouse *IGF2/Igf2* can be expressed from several different promoters, but in the mouse, the transcripts from one promoter—*Igf2P0*—are placental-specific [51]. Deletion of the P0 promoter reduces placental size close to that of complete *Igf2* KO, i.e., around 40% smaller than normal [51]. Humans also have an *IGF2* P0 promoter, but it is not placental-specific, indicating only a partial conservation of imprinting of *IGF2P0/Igf2P0* between mice and humans [52].

The main role of the placenta is the nutrition of the fetus. Murine *Igf2P0* transcripts are expressed specifically in the labyrinthine trophoblast of the placenta [51], the cellular interface between the maternal blood supply and the fetal capillaries, and the surface across which nutrient exchange with the fetus takes place. Through increasing the surface area, *Igf2P0* is thought to enhance passive permeability in the labyrinth, promoting nutrient exchange [51], [53]. In the *Igf2P0*-null model, fetal *Igf2* expression is shown to regulate nutrient supply from the growth-restricted placenta in a paracrine manner [51]. The presence of circulating fetal Igf2 coincident with an imbalance between placental supply and fetal demand results in upregulation of nutrient transfer systems [54]. Placental transcription of glucose transporter *Slc2a3* and paternally expressed amino acid transporter *Slc38a4* are upregulated, followed by an increase in glucose and amino acid transport from the placenta to the fetus [54]. These data show that imprinted growth regulators may influence nutrient supply through action in the placenta, or by regulating demand from the fetus.

As previously discussed, the expression level of *PHLDA2/Phlda2* correlates inversely with fetal growth in both humans and mice [33], [41], [55]. This role as a growth suppressor has recently been directly linked with the exchange of nutrients between mother and fetus in mice [56]. In a transgenic model, a two-fold increase in *Phlda2* expression resulted in reduced placental weight, specifically in the junctional zone, and a decrease in glycogen stores and glycogen cell migration, important for fetal glucose supplies late in gestation [56]. This is the reverse of what is seen in the *Phlda2* knockout mouse, and unlike the null, impacted on embryonic as well as placental development so that overexpression of *Phlda2* led to 13% reduction in fetal weight [55], [56]. These data suggest that the regulation of fetal and placental growth by *PHLDA2/Phlda2* might be effected through its potential role in nutrient transfer [56].

## The *KCNQ1/Kcnq1* Imprinting Cluster

**Figure 2:**
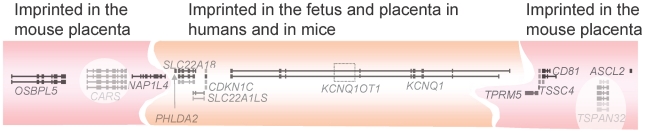
The *KCNQ1/Kcnq1* imprinted gene cluster. The *KCNQ1/Kcnq1* region on human Chromosome 11/mouse Chromosome 7 is the largest known imprinting cluster in mice. The central *KCNQ1OT1* transcript is paternally expressed and executes silencing of the other transcripts on this allele, so the rest are maternally expressed only. The region is smaller in the mouse fetus than the placenta, and this feature is not conserved in the human where the flanking transcripts are universally biallelic. *CARS* and *TSPAN32* are not imprinted and are shown with a white back ground to reflect this.

Expression within the *KCNQ1/Kcnq1* imprinting cluster on Hsa11/Mmu7 is only partially conserved between humans and mice [25]. Whilst the central six transcripts, covering 400 kb, maintain monoallelic expression in both species, the eight flanking genes are known to be maternally expressed in the mouse and bovine placenta, extending the imprinted domain to 780 kb [57]–[59]. In contrast, these flanking transcripts are biallelic in the human [25] (Figure 2).

The function of several of the genes in the *KCNQ1/Kcnq1* cluster has been extensively characterised, and correspond with roles in embryonic and placental growth. *ASCL2/Ascl2* is essential for early placental development, whilst *CDKN1C/Cdkn1c* is a growth suppressor, whose absence causes neonatal lethality in the mouse [60], [61]. Mutations or epimutations affecting *CDKN1C* result in one type of BWS in the human, commonly involving severe abdominal wall defects [62], [63]. Another group of BWS cases are due to mutations or epimutations immediately upstream of *H19*. These BWS patients have a high risk of tumours, especially compared to the *CDKN1C* region (epi)mutation group; see Table 1 [63]. *KCNQ1* is imprinted at the majority of expressed sites in the human, except in the heart, the site of the defect in long-QT syndrome that are caused by mutations in *KCNQ1* [64], [65]. *PHLDA2*, whose role has already been discussed, is also encoded at this locus.

A differentially methylated region (DMR) in intron 10 of *KCNQ1* acts dually as the imprinting control region (ICR) for the cluster, called KvDMR, and the promoter of an antisense ncRNA *KCNQ1OT1*, which contributes to the regulation of imprinting at the domain [66], [67] (Figure 2). In the mouse, this ncRNA is imprinted and expressed from the paternally inherited chromosome where its transcription is required for the repression of the paternally inherited protein coding genes in cis [68], [69]. *Kcnq1ot1* RNA may be linked to recruitment of the Eed-Ezh2 polycomb protein complex to the paternal chromosome, resulting in the enrichment of H3K27Me3 and H3K9Me2 and a repressed chromatin conformation conducive to allelic silencing [58]. *Dnmt1−/−* mice are deficient in DNA methytransferase DNMT1, the enzyme responsible for maintenance of DNA methylation. In these mice, histone modifications are able to maintain imprinting of the placental specific genes in the *Kcnq1* region, indicating that maintenance methylation is not required for prolonged monoallelic expression of these genes in the placenta. Imprinting of the central six genes is lost in *Dnmt1−/−* mice [57], [58], [70], indicating that they do require maintenance methylation for monoallelic expression. Despite the absence of a requirement for maintenance methylation for the imprinting of a subset of genes in this cluster, the establishment of the germline DMR (by different enzymes, the *de novo* DNA methyltransferase, *Dnmt3a* [71]) remains essential for imprinting across the whole locus [70]. There is evidence that the murine *Kcnq1ot1* RNA may form a silencing compartment in the nucleus, to which the repressed alleles are localised [72]. This compartment is larger in the murine placenta than in the fetus, perhaps reflecting the increased size of the repressed region in this tissue [73]. Given that imprinting of the *KCNQ1* region in the human embryo and placenta both mirror that of the mouse embryo, if this model is correct it may be that such a transcriptional silencing compartment would be smaller in the human placenta, encompassing only the central seven transcripts.

## Differences in the Placenta Between Humans and Mice

The placenta is the organ with the most varied morphology between mammalian species [74]. This is indicative of the different reproductive strategies employed by different species, where young may be precocial or altricial, and litter size and gestational length vary greatly. The lack of conservation of imprinting between humans and mice in the placenta, such as that of *IGF2PO* and the *KCNQ1* region, has been suggested to be due to the marked differences between murine and human placentation and pregnancy [75], [76]. Mice have a labyrinthine interdigitation into the maternal decidua, compared to the villous structure of the exchange surface in the human. Mouse placentas have one or very few central maternal arteries, but in the human, several maternal spiral arteries provide the placenta with nutrients and oxygen. In the mouse, glycogen cells in the placenta become abundant between E13 and E18.5, invade the decidua basalis, and cluster at the base of the central maternal artery. They lyse just before term, possibly to provide energy for the final phase of prenatal growth [77]. Both species manipulate the maternal blood supply to maximise nutrient transfer. In the mouse, it is suggested that the primary cause of maternal artery transformation is the secretion of cytokines—i.e., by glycogen cells, which secrete Igf2 protein and express nuclear *Cdkn1c* and have been shown to be important for transformation of the central maternal artery [78], [79]. Artery transformation in the mouse is shallow and limited to the proximal decidua [75]. Conversely, human maternal arteries are extensively colonised by endovascular trophoblast cells. These cells relax the elastic artery walls and expand the lumen, allowing increased blood flow to the growing human fetus.

## Differences in Imprinted Gene Expression Between Human and Mouse Placentas

In the mouse, 5–15 fetuses may be carried in utero at the same time, depending on the mouse strain, and one pregnancy can occur from two separate matings [80], [81]. This intra-brood competition forms the basis of one of the key features of the parental conflict theory because such a scenario would be predicted to increase parental conflict at the materno-placenta interface [82]. Different levels of conflict in the placenta between mice and humans may account for the divergence in imprinted gene expression profiles, with imprinted expression of certain genes not being required in the human. The transcriptional regulator *Ascl2* is imprinted in the mouse placenta, and absolutely required for placentation, whereas in the human this gene is biallelically expressed, indicative of a less stringent requirement for dosage management in humans for this gene [60], [83], [84], or the utilisation of a different mechanism of dosage control in the human. Sheep, like humans, bear singletons and the sheep orthologue of placental specific *Ascl2 (SASH2)*, is biallelically expressed whilst *CDKN1C* is maternally expressed [85]. To date, most genes that are imprinted in mouse but not in human, including those previously discussed, are imprinted specifically in the placenta of the mouse. Table 2 lists placental-specific imprinted genes identified in the mouse at several loci. With the exception of *TFPI2* these genes are not imprinted in the human [25]. This observation lends support to the idea that the placenta could be at the centre of the differences in imprinting between mice and humans. Of the genes listed in Table 2, most are maternally expressed, consistent with an involvement of these genes in limiting placental and/or fetal growth [25]. Perhaps the mouse placenta manages parental conflict through a more limited invasion of the maternal decidua and blood vessels than that of the human placenta, with imprinted genes playing a role in modulating the process.

**Table 2:** Conservation of imprints in human and mouse http://igc.otago.ac.nz.

Gene (murine notation)	Mouse	Human
*Gatm*	Imprinted in placenta	Not imprinted
*Igf2r*, *Air*	Imprinted	Not imprinted/No orthologue
*Pon 2*, *3*, *Asb4*,	Imprinted in placenta	Not imprinted
*Nap1l4*, *Osbpl5*, *Cd81*, *Ltrpc5*, *Tssc4*, *Ascl2*	Imprinted in placenta	Not imprinted
*Ampd3*, *Th*, *Dhcr7*	Imprinted in placenta	Not imprinted
*Dcn*	Imprinted in placenta	Not imprinted
*Slc22a2*, *Slc22a3*	Imprinted in placenta	Not imprinted (*SLC22A2* polymorphic)
*Xist/Tsix*	Imprinted in placenta throughout gestation, and in preimplantation embryo	Not imprinted

Many genes in the mouse are imprinted specifically in the placenta. A lack of conservation exists between the human and the mouse, where these placental-specific genes are not imprinted at all in the human. Of these genes, all except *Air* and *Tsix* are maternally expressed, and except *Igf2r*, *Air* and *Ascl2*, are all confined either in their expression or their imprinting to the placenta [25], [46], [52], [104].

Total reproductive capability of mammalian females over a lifetime could also have an impact on parental conflict, and so possibly imprinted gene expression, since deleterious effects of pregnancy on the mother may be additive between pregnancies. It would therefore be illuminating to compare imprinting in monoseasonally oestrous species, such as the giant panda, with imprinting in mammals capable of many fertile oestrous cycles in their lifetimes, such as mice and humans.

## Changes in Global Gene Expression in the Placenta during Gestation

Genome-wide expression analyses of early and late murine and human placentas show that early placentation events are more similar between mammalian species than later placental growth [86]. During early gestation and placental developmental stages—i.e., E8.5 to E10.5 in the mouse—the placenta utilises evolutionarily ancient genes, such as those involved in metabolism, the cell cycle, and RNA processing. During mid to late gestation (E10.5 to E15) a transition occurs where expression profiles become enriched for genes that evolved since the divergence of rodents and primates from their common ancestor. In rodents, from E15 to P0 genes specific to the rodent placenta are expressed, and in the human placenta primate-specific genes are all enriched compared to the mouse [86].

This striking selection for high expression of evolutionarily new, species-specific genes, during mid-gestation with specificity increasing as gestation continues, is indicative of the progressive divergence of placental physiology during development. Concomitantly, the conservation of genomic imprinting between humans and mice may be dynamic through pregnancy. Imprinting can be developmentally regulated by epigenetic regulators that are tissue-specific. Germline methylation can therefore be “read” differently in different cell types and at different stages in development, resulting in, for example, the highly tissue-specific imprinting at the *GNAS* locus on Hsa20/Mmu2 [87]. Differential reading of the germline methylation mark could depend on the presence of tissue-specific transcription factors or epigenetic effectors such as polycomb group proteins. For example, allelic histone modifications in the *Kcnq1* region are required to maintain imprinting of placental-specific imprinted genes in the mouse placenta and are able to do so without maintenance of differential methylation at the KvDMR, which is not the case for the genes imprinted in the embryo that still require an intact KvDMR [57], [58]. As placental physiology diverged throughout gestation, differences in developmentally regulated imprinting may also have evolved. It is possible that placental-specific imprinting seen in the mouse (Table 2) may be present in the human placenta, but at a much earlier gestation than has so far been analysed, before differentiation resulted in biallelic expression of these genes. Similarly, in later gestation in humans, genes not imprinted in the mouse may be imprinted in the human placenta.

## Imprinting in the Postnatal Human

After birth, resource allocation is distinct from that during pregnancy, and the interaction between offspring and mother is vastly changed. The placenta, and its function to transfer nutrients from the maternal bloodstream and pass them on to the fetus, is no longer present, and the neonate has developed strategies to function ex utero, leading to full independence after weaning. Key organs for imprinted expression postnatally include the brain and endocrine tissues, such as brown adipose tissue, which regulates non-shivering thermogenesis, a pre-weaning postnatal adaptation to independent life [88]. Genes whose imprinted expression was previously vital in the placenta, may cease to be important in some tissues, exemplified by the biallelic expression of *IGF2* in human adult liver [89].

**Figure 3:**
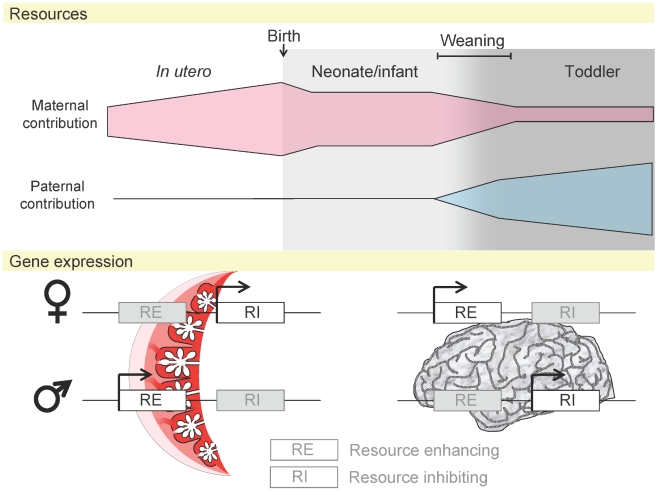
Maternal and paternal resource allocation before and after birth. Before birth and during weaning the mother's role in fetal nutrition and support far outweighs the father. Following weaning the role of the father increases. The placenta is only involved in utero; following this, the brain is likely to be the organ most important in the drive for resources. Expression of imprinted genes, acting as resource enhancers (RE) or inhibitors (RI), may alter to reflect this [92].

It is likely that parental conflict in mammals therefore continues after birth, albeit in an altered fashion (Figure 3) [90]. During the period between weaning and independence of children from their parents, the father has an increased role given his position as “breadwinner” that may be an investment of higher magnitude than that of the mother in older children [91], [92]. Postnatally, some aspects of several imprinting syndromes seem incompatible with the conflict theory in its simplest form. For example, PWS results from a loss of paternally expressed transcripts, yet PWS children are characteristically large. This can be reconciled with the concept of resource allocation by focussing on behaviour. Genes imprinted in the PWS/AS region, which are highly expressed in the brain, may act postnatally to modify behaviour to maximise resources (Figure 3). Emotional and behavioural cues could be utilised by the neonate to manipulate parents in order to provide adequate nutrition. In AS, caused by loss of expression of maternally expressed *UBE3A*, children prolong suckling and exhibit convivial behaviour that maximises maternal input [93]. In PWS, resulting from the loss of paternally expressed HBII-85 snoRNAs, children suckle badly and wean quickly but are hyperphagic after birth, arguably maximising utilisation of paternal resources and minimising usage of maternal ones [94], [95]. So, conflict exists after birth, but its arena might be considered to have moved from the placenta to the brain [92]. Whether this facet of imprinting displays consistency between humans and mice remains to be seen. Mouse models with targeted deletions of the MBII-85 snoRNA cluster display characteristic PWS features of hypotonia and a failure to thrive, followed by hyperphagia [96], [97]. The mice do not become obese, indicating some species-specific differences in metabolism, however the behavioural parallels between between PWS and the MBII-85-deleted transgenic mouse indicate that some aspects of postnatal conflict may be managed similarly between the two species [96], [97]. Imprinting in the brain is conserved between mice and humans at the *GRB10/Grb10* locus, where transcripts are paternally expressed in the central nervous system through similar tissue specific chromatin modifications [46], [98]–[100]. *Grb10* is a growth inhibitor and is maternally expressed in most tissues in mouse [49]. In utero, *Grb10* negatively regulates fetal and placental growth, whilst it is involved in glucose homeostasis in adult muscle and adipose tissue [101]. The function of *Grb10* in brain and the purpose of its maternal suppression is unknown. The distinct mechanism of *GRB10/Grb10* regulation observed in human and mouse brain [46], [100], and opposing allelic repression compared to other tissues, is suggestive of it having a distinct role in this tissue, perhaps in influencing postnatal behaviour in the father's favour [46]. Imprinting in the brain is a developing field, one that will provide new and exciting insights into human behaviour and the evolution of imprinting.

## Summary

The biological function of reducing the diploid state to functional haploidy has to be questioned in terms of its evolutionary significance. A case needs to be made for the benefit of silencing of one parental allele balanced against the negative impact of a mutation at the remaining allele that would leave the cell with no gene product.

In humans, inappropriate expression of imprinted genes leads in many cases to severe syndromes. This shows that the monoallelic expression of this small subset of genes is indispensible for normal human development. Aberrant prenatal growth occurs frequently in imprinting syndromes. This shows that an important feature of imprinting is the regulation of growth and nutrient transfer between mother and fetus, for which the placenta is key. This regulation should be balanced to serve the interests of both parents equally.

There are several genes that are imprinted in mice but not in humans. This is suggestive of a difference in importance or function of these transcripts between these two species, possibly due to species-specific differences in their respective placental physiology. The lack of conservation in imprinted expression for some genes may also be linked to a reduction in conflict during human pregnancy compared to the mouse, as humans bear singletons rather than large litters, and so have little or no possibility of multiple paternity.

Whilst differences in the reproductive biology of mice and humans are evident, large distinctions in imprinting in organs unrelated to pregnancy have not yet been identified. Following birth, offspring are free from maternal growth constraints, no longer rely on the placenta and must now recruit input from both parents in order to maximise fitness. A resolution of parental conflict postnatally will therefore rely on specific behavioural and emotional cues, engaging organs such as the brain and endocrine axis.

Genomic imprinting in humans is clearly important. Analysis of imprinting disorders and information from closely related mammalian models allows us to define the importance of its conservation and the relevance of any absence of conservation. Through further focussed research into human imprinting, we will elucidate the specialised functions of this remarkable transcriptional mechanism in our species.
